# Oxytocin receptor disruption in *Avil-*expressing cells results in blunted sociability and increased inter-male aggression

**DOI:** 10.1371/journal.pone.0260199

**Published:** 2021-11-30

**Authors:** Manal Tabbaa, Ashley Moses, Elizabeth A. D. Hammock

**Affiliations:** Department of Psychology and Program in Neuroscience, The Florida State University, Tallahassee, FL, United States of America; California State Polytechnic University Pomona, UNITED STATES

## Abstract

Social behaviors are foundational to society and quality of life while social behavior extremes are core symptoms in a variety of psychopathologies and developmental disabilities. Oxytocin (OXT) is a neuroactive hormone that regulates social behaviors through its receptor (OXTR), with all previously identified social behavior effects attributed to the central nervous system, which has developmental origins in the neural tube. However, OXTR are also present in neural crest-derived tissue including sensory ganglia of the peripheral nervous system. *Avil* encodes for the actin-binding protein ADVILLIN, is expressed in neural crest-derived cells, and was therefore used as a target in this study to knock out OXTR expression in neural-crest derived cells. Here, we tested if OXTRs specifically expressed in *Avil* positive neural crest-derived cells are necessary for species-typical adult social behaviors using a Cre-LoxP strategy. Genetically modified male and female mice lacking OXTR in *Avil* expressing cells (OXTR^*Avil*^ KO) were tested for sociability and preference for social novelty. Males were also tested for resident intruder aggression. OXTR^*Avil*^ KO males and females had reduced sociability compared to OXTR^*Avil*^ WT controls. Additionally, OXTR^*Avil*^ KO males had increased aggressive behaviors compared to controls. These data indicate that OXTRs in cells of neural crest origin are important regulators of typical social behaviors in C57BL/6J adult male and female mice and point to needed directions of future research.

## Results & discussion

A diverse body of evidence supports a role for the oxytocin (OXT) and the OXT receptor (OXTR) in modulating the brain and social behaviors across species [1, 2]. Studies in mice testing the necessity of OXTR in regulating social behavior have compared the social behaviors of congenital OXTR knock-out (OXTR KO) mice to wild-type (OXTR WT) mice [3–7]. Sociability (preference for a mouse more than an object), social novelty (preference to investigate a new mouse more than a familiar mouse), and resident intruder (RIT) aggression tests have been used to investigate the social phenotype of OXTR KO mice while interacting with same-sex conspecifics. OXTR KO mice show reduced sociability and reduced preference for social novelty compared to OXTR WT controls [3, 4, 6, 7]. Moreover, male OXTR KO mice have consistently displayed a higher aggressive phenotype compared to OXTR WT males in the RIT [4–7].

Studies focusing on site-specific knock out of the OXTR thus far indicate dissociable roles of OXTR in sociability, social novelty preference, and aggression [8–10]. However, all prior research into the site-specific roles of OXTR in regulating social behaviors has focused on brain mechanisms. Significantly, OXTR in the neural crest-derived peripheral nervous system (PNS) of developing mice has recently been characterized [11, 12]. Moreover, topical oral administration of OXT was recently reported to regulate sensory dependent brain activity and behavior in developing mice suggesting a functional neurobehavioral role for peripheral OXTR [13]. Further, intranasal OXT and peripheral injections of OXT have brain and behavioral consequences [14–18], which may be due to peripheral OXTR activation including reciprocal interactions with brain OXTR. In this study, we tested the consequences of OXTR loss specifically from *Avil*-expressing cells, which are neural-crest derived cells primarily located in the PNS, on adult social behavior in male and female C57BL/6J mice. *Avil* encodes the actin-binding protein ADVILLIN and is expressed in all dorsal root ganglion neurons, the trigeminal ganglion, enteric, parasympathetic and sympathetic neurons as well as their peripheral projections, particularly projections innervating blood vessels and Merkel skin cells [19]. In addition, ADVILLIN is expressed in uterus, intestines, and taste buds as well as in the brain at low levels in the dorsal habenula and mesencephalic trigeminal nuclei of the brainstem [19].

**Fig 1:**
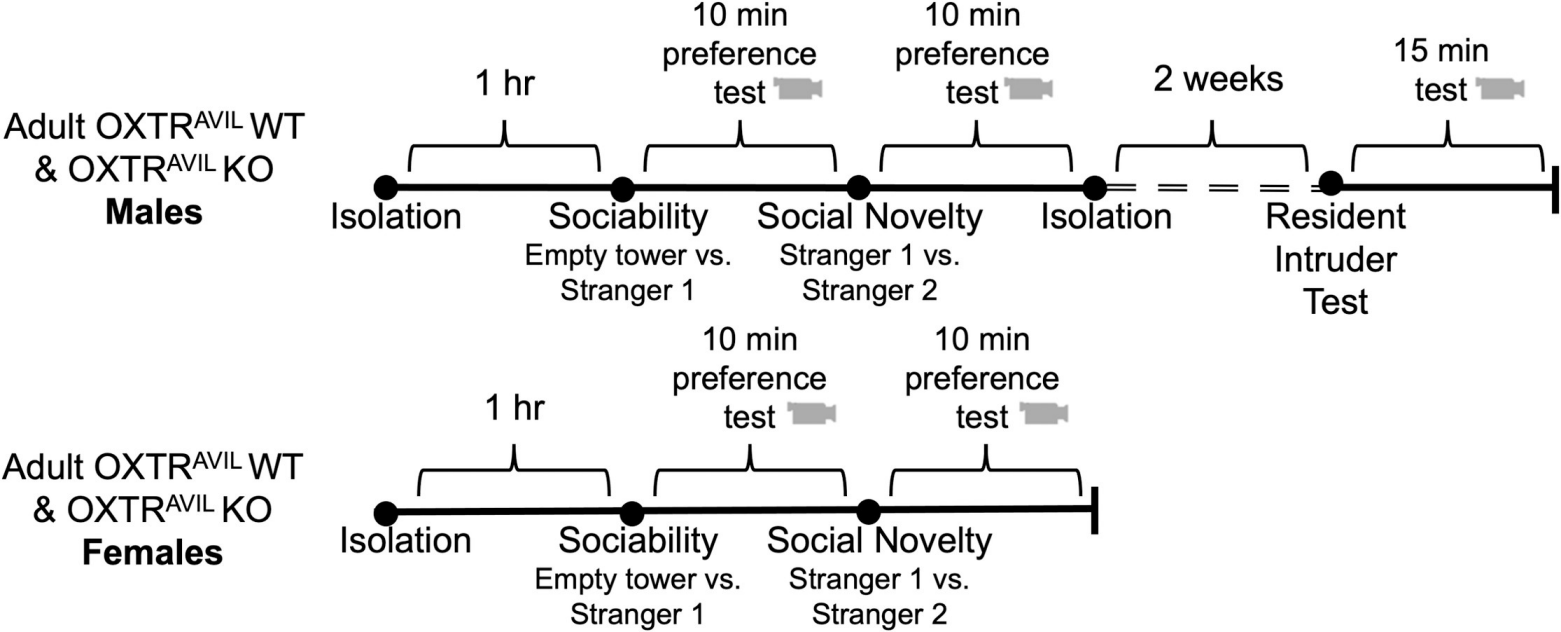
Experimental timeline. OXTR^*Avil*^ WT (*fxOxtr*^*fx/fx*^; *Avil*-Cre-) and OXTR^*Avil*^ KO (*fxOxtr*^*fx/fx*^; *Avil-Cre+*) males (top) and females (bottom) were isolated for 1 hour prior to sociability testing in the three chambered apparatus. The social novelty test took place directly after the sociability test and both tests were 10 minutes each. Directly after the social novelty test, males were single housed for at least 2 weeks and then tested for aggression in the resident intruder test which lasted for 15 minutes. All behavioral tests were video recorded.

To generate mice with OXTR deleted specifically from *Avil* expressing cells, we used a Cre-LoxP strategy in well-established lines of mice to generate OXTR^*Avil*^ WT and OXTR^*Avil*^ KO subjects. OXTR^*Avil*^ KO and WT adult male and female mice were tested for sociability and social novelty preference, and males were tested for aggression (Fig 1). Using RT-qPCR, we confirmed the loss of *Oxtr* mRNA expression in peripheral tissue and intact *Oxtr* mRNA expression in brain tissue in adult OXTR^*Avil*^ KO mice (S1 Fig in S1 File).

**Fig 2:**
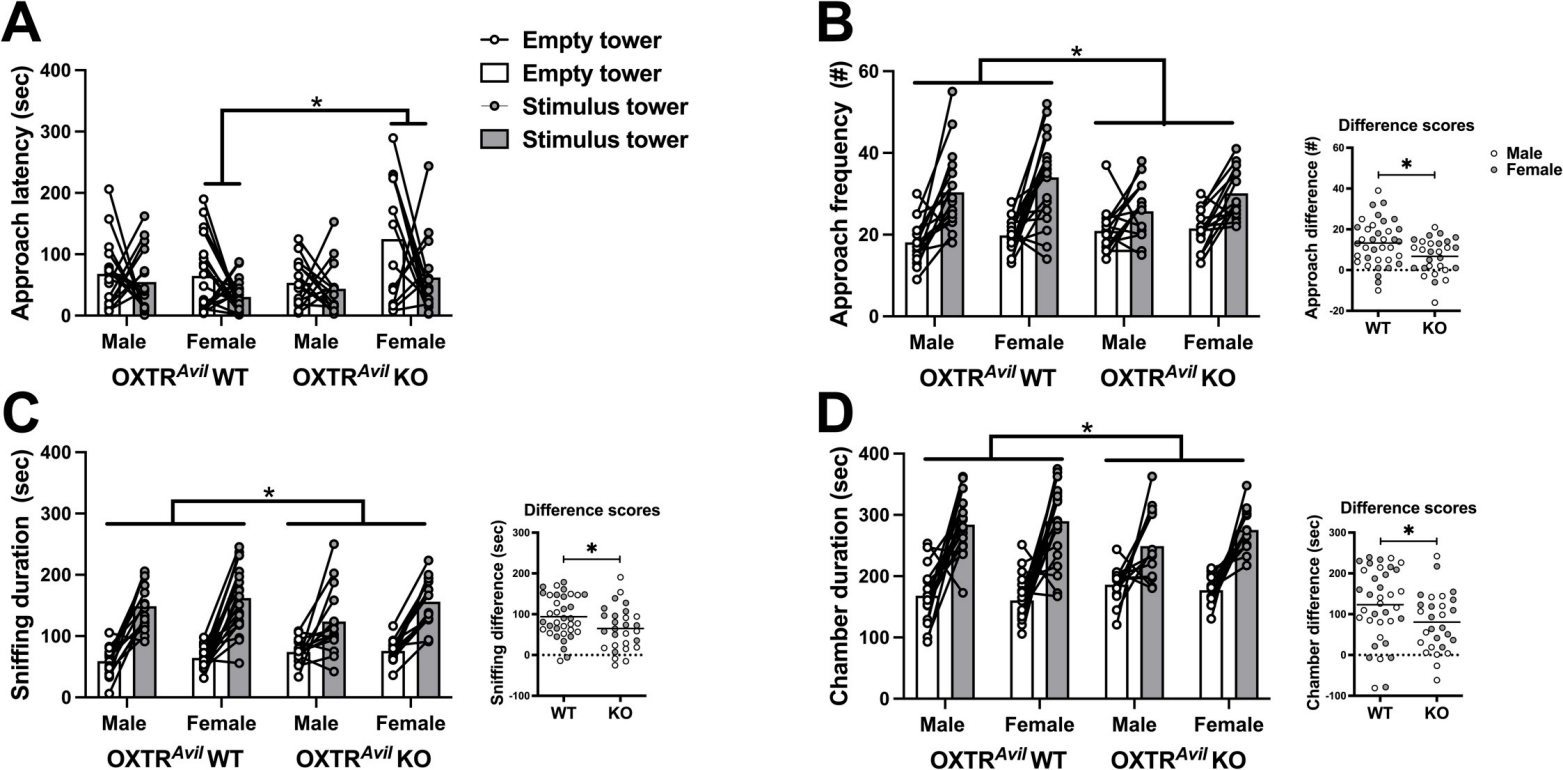
Sociability is present but significantly blunted in male and female OXTR^*Avil*^ KO mice compared to WT. Sociability—the preference for a holding tower containing a same-sex stimulus mouse (stimulus tower; gray markers) over an empty tower (empty tower; white markers)—is evident across groups by a decreased latency to approach the stimulus tower (A; F_1, 60_ = 4.934, p < .05; d = 0.57) and increased approach frequencies (B; F_1, 60_ = 57.774, p < .001; d = 1.98), sniffing durations (C; F_1, 60_ = 148.166, p < .001; d = 3.14), and chamber durations (D; F_1, 60_ = 94.729, p < .001; d = 2.51) of the stimulus tower compared to the empty tower. OXTR^*Avil*^ KO females had increased latencies to approach the towers compared to OXTR^*Avil*^ WT females (A; F_1, 60_ = 13.643, p < 0.001). OXTR^*Avil*^ KO males and females had reduced approach frequencies (B; F_1, 60_ = 6.336, p < .05; d = 0.65), investigation (sniffing) durations (C; F_1, 60_ = 4.896, p < .05; d = 0.57), and chamber durations (D; F_1, 60_ = 4.067, p < .05; d = 0.52) between the conspecific containing tower/chamber and empty tower/chamber compared to OXTR^*Avil*^ WT males and females. Data are graphed as individual data points and group averages. Difference scores (inset graphs) were calculated by subtracting approach frequency and sniffing/chamber duration of the empty side from the side containing the stimulus mouse. *in A = between-subject genotype by sex interaction, adjusted p < 0.05; * in B-D = within-subject genotype by tower type interaction, p < 0.05. See also S2 Fig in S1 File, S1 Table in S1 File.

First, we evaluated sociability behaviors including latency to approach the tower containing a novel social stimulus mouse and an empty tower, frequency and duration of investigating both towers, and chamber durations. As expected, there was a significant within-subject preference for the social stimulus tower compared to the empty tower on all four measures (Fig 2, S1 Table in S1 File).

In addition to strong evidence of the presence of sociability in this cohort of mice, there was an interaction between OXTR^*Avil*^ genotype and tower type demonstrating reduced approach frequencies, tower investigations, and chamber durations in OXTR^*Avil*^ KO mice (Fig 2, S1 Table in S1 File). As depicted in Fig 2, male and female OXTR^*Avil*^ KO mice had lower sociability difference scores than WT mice. In addition, a genotype by sex interaction between subjects followed up with Bonferroni corrected sex comparisons revealed OXTR^*Avil*^ KO females took longer to approach the towers compared to OXTR^*Avil*^ WT females. The blunted sociability in OXTR^*Avil*^ KO mice could be due to the loss of *Oxtr* in *Avil*-cre expressing cells or the presence of the Cre transgene. To control for this, we ran a separate cohort of mice with and without the *Avil*-Cre transgene. Importantly, the *Avil*-Cre control experiment also showed robust evidence of sociability (significant main effect of tower type) but no effect of the Cre transgene on sociability (no genotype x tower type interaction; S2 Fig in S1 File, S1 Table in S1 File), confirming that it is the loss of *Oxtr* expression in *Avil* expressing cells and not the presence of the Cre transgene driving the reduction in sociability in OXTR^*Avil*^ KO mice.

**Fig 3:**
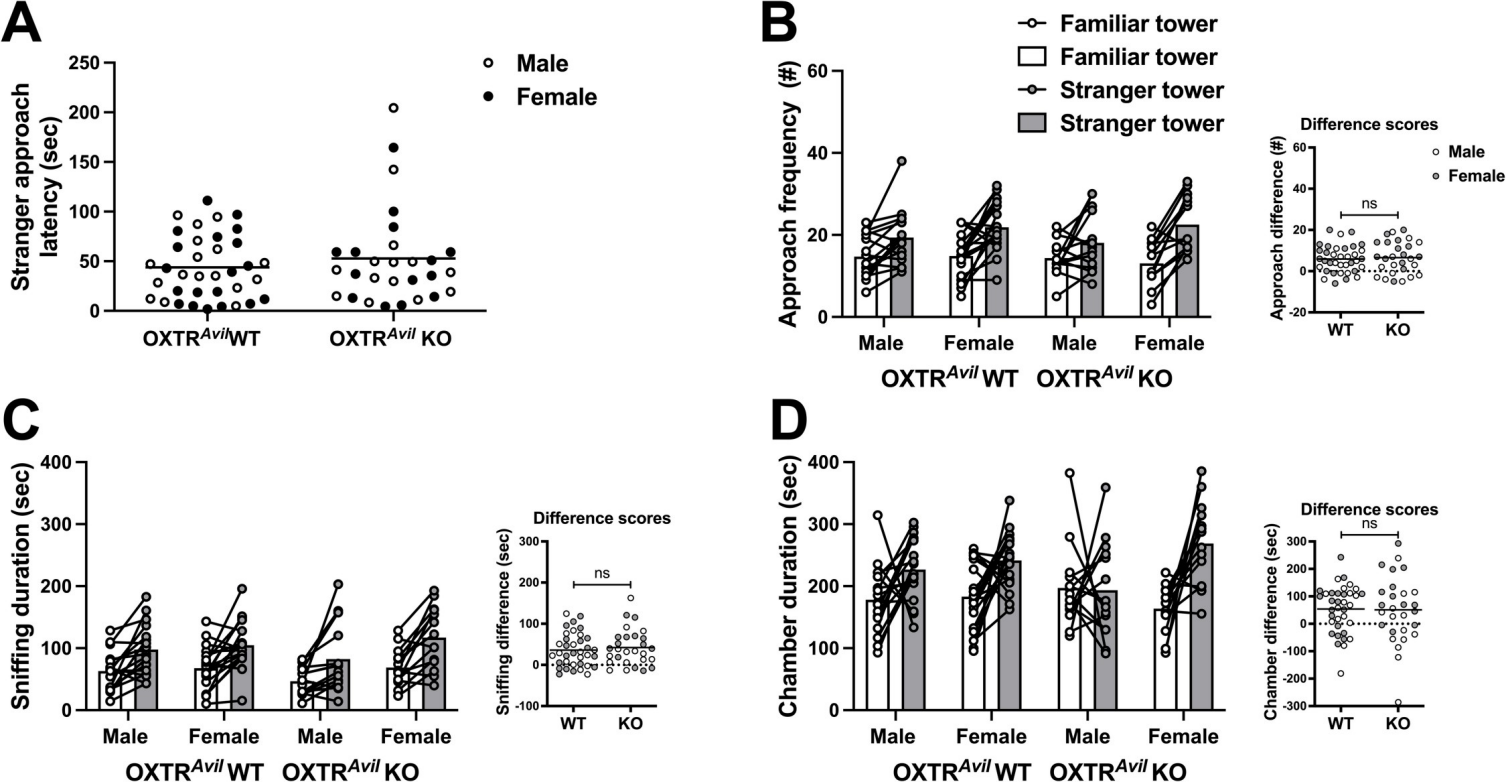
Preference for social novelty behaviors were similar in OXTR^*Avil*^ WT and KO males and females. OXTR^*Avil*^ mice displayed a preference for social novelty—a preference for a tower holding a novel stranger mouse (stranger tower; gray markers) over a tower holding a familiar mouse (familiar tower; white markers)—as evident by increased approach frequencies (B; F_1, 60_ = 51.23, p < .001; d = 1.85) to the stranger tower, increased sniffing (C; F_1, 60_ = 52.208, p < .001; d = 1.864), and chamber durations (D; F_1, 60_ = 17.855, p < .001; d = 1.09). Social novelty preference was not impacted in OXTR^*Avil*^ KO mice (no significant genotype x tower type interaction). Data are graphed as individual data points and group averages. Difference scores were calculated by subtracting approach frequency and sniffing/chamber duration towards the familiar conspecific from the stranger conspecific. ns = not significant. See also S3 Fig in S1 File, S1 Table in S1 File.

After evaluating sociability, we evaluated preference for social novelty. As expected, the OXTR^*Avil*^ cohort showed strong evidence of preference for social novelty including increased approach frequencies, sniffing, and chamber durations for the tower containing the stranger mouse compared to the familiar mouse (Fig 3, S1 Table in S1 File). Unlike sociability, social novelty preference was not impacted in OXTR^*Avil*^ KO mice (no significant genotype x tower type interaction, Fig 3, S1 Table in S1 File). Accordingly, the *Avil*-Cre control cohort showed significant social novelty preference and no Cre genotype effects on social novelty preference (S3 Fig in S1 File, S1 Table in S1 File).

Previous studies have documented a large effect size of reduced sociability in conventional adult OXTR KO males, compared to WT (d = 1.2–1.8) [3, 6, 7], while the effect size of reduced sociability in the OXTR^*Avil*^ KO males in our study was medium (average d = 0. 71). We tested both males and females and found no sex-specific effect on sociability (male and female average d = 0.58). Thus, while whole body OXTR loss impacts adult sociability to a greater extent than just neural-crest derived OXTR loss, life-long loss of OXTR in *Avil* cells is sufficient to disrupt sociability levels in males and females and may account for half of the impaired sociability reported previously in congenital OXTR KO male mice. Other studies that have used Cre strategies to identify a role for brain OXTR in sociability have not observed a disruption with brain specific OXTR loss. For example, selective deletion of OXTR from *CaMKIIα* -expressing neurons [20] or by viral targeting the lateral septum [21] or the hippocampus [22] did not impair sociability.

OXTR in *Avil* expressing cells may be specific to regulating sociability levels but might not be involved in preference for social novelty or its social recognition prerequisite. In contrast, brain OXTR do seem to be needed for these behavioral components as Cre-based deletion of OXTR from the lateral septum does impair social novelty preference [21]. Considering that whole-body OXTR KO reduces social novelty preference in adult mice and prairie voles [6, 7, 23], but we did not observe this effect in OXTR^*Avil*^ KO mice, suggests that central OXTR but not neural-crest derived OXTR are necessary for the behavioral display of adult social novelty preference.

Reduced sociability in OXTR^*Avil*^ KO adults may be a result of a developmental and/or activational effect. Future experiments involving temporal regulation of OXTR in sensory ganglia will resolve this question for adult sociability. While we did not observe differences in social novelty preferences in adults, differences might be present in younger ages. Future studies can address this by testing males and females for sociability and the developmental shift in preference for familiarity to social novelty.

Compensatory mechanisms may have blunted or negated potential effects of the absence of OXTR in *Avil* cells on social behaviors. Because OXT injections rescue sociability and preference for social novelty deficits in congenital OXTR KO males [6] and OXT can bind to vasopressin (AVP) receptors, AVP receptors may compensate for peripheral OXTR loss. Indeed, central AVP receptors are involved in sensory processes, including olfaction and social novelty preference [6, 24]. This could function through known brain pathways or through peripheral sensory ganglia. AVP receptor 1a (*Avpr1a*) mRNA is present in sensory ganglia and receptor binding sites are present in various peripheral tissues of neonatal mice [25, 26].

The contribution of OXTR in *Avil* expressing cells to reduce sociability in mice is reminiscent of mouse models of neurodevelopmental disorders which also exhibit reduced sociability that has been attributed to altered tactile discrimination and developmental loss of the autism spectrum disorder (ASD)-associated genes, *Mecp2* or *Gabrb3*, in *Avil* cells [27]. Blunted OXTR signaling in *Avil* cells may interact with ASD-associated genes during development to contribute to ASD core symptoms and subtypes. Peripheral changes alone may hinder the sensory-dependent development of forebrain regions involved in regulating adult social behavior, consistent with a developmental diaschisis model of neurodevelopmental disorders [28].

**Fig 4:**
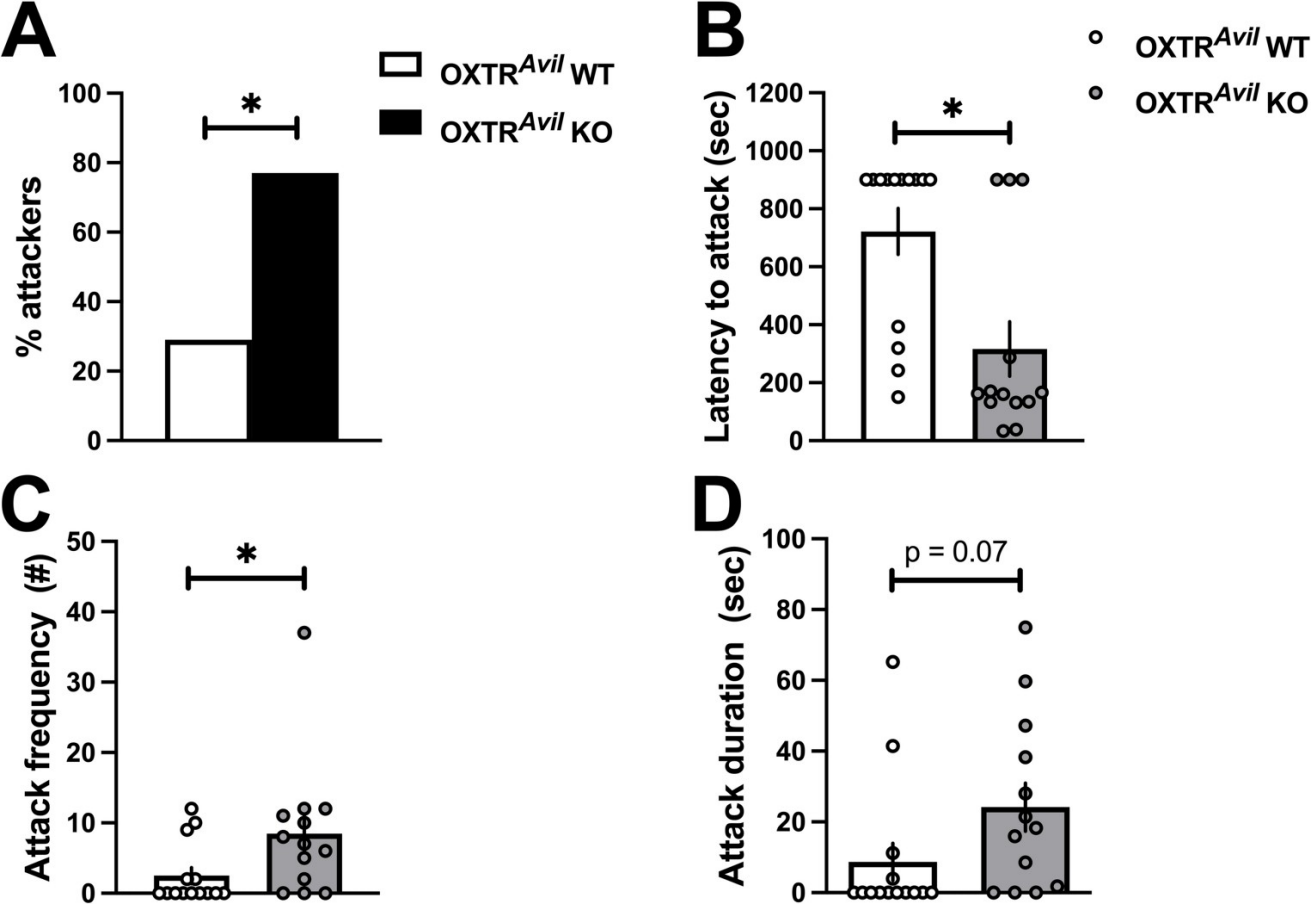
Increased aggression in OXTR^*Avil*^ KO males compared to OXTR^*Avil*^ WT males in the resident intruder test (RIT). A higher percentage of OXTR^*Avil*^ KO males (black bar) displayed aggression in the RIT compared to OXTR^*Avil*^ WT males (white bar, A). OXTR^*Avil*^ KO males (gray markers) also had reduced latencies to fight (B; F_1, 27_ = 11.862, p < .01; d = 1.27) and increased frequencies of attack (C; F_1, 27_ = 4.719, p < .05; d = 0.83) compared to OXTR^*Avil*^ WT males (white markers). Duration of fighting was not significantly different between OXTR^*Avil*^ WT and KO males (D). Frequency and duration are graphed as individual data points and group averages ± standard error of the mean. A latency of 900 seconds was assigned to subjects that did not attack during the 15-minute-long test. See also S4 Fig in S1 File, S1 Table in S1 File.

After measuring sociability and social novelty preference, males were isolated for two weeks and tested in the RIT. More OXTR^*Avil*^ KO males initiated aggression than OXTR^*Avil*^ WT males and had shorter clinch attack latencies and higher number of attacks compared to WT (Fig 4, S1 Table in S1 File). The effect of genotype on fighting durations was not significant, and there were no differences in other non-aggressive behaviors between OXTR^*Avil*^ WT and KO males including social investigation durations, frequencies, and non-social exploratory behaviors. The *Avil*-Cre transgene alone did not explain increased aggression in OXTR^*Avil*^ KO mice (S4 Fig in S1 File, S1 Table in S1 File).

The magnitude of the effect of OXTR^*Avil*^ KO on aggression in our study (average Cohen’s d ~1.1) is similar to several studies using congenital OXTR KO mice (estimated Cohen’s d ~ 1.2) [4–7]. This suggests that loss of OXTR in neural crest derived cells are the primary contributor to the aggression phenotype in conventional OXTR KO mice.

The literature on OXTR in aggression hints strongly at a developmental role for OXTR. Dhakar et al., (2012) found increased aggression in congenital OXTR KO compared to OXTR WT mice in the RIT [5]. Surprisingly, a brain-specific post-weaning knockout of OXTR using a Cre transgene under the control of the *CaMKIIα* promoter did not show an aggression phenotype. They concluded that OXTR in the pre-weaning stage is important in the development of typical aggression. Additionally, Takayanagi et al., (2005) reported heightened aggression in the RIT by OXTR KO and OXT KO males [4]. However, the aggression phenotype of the OXT KO depended on the genotype of the dam: OXT KO males born to an OXT KO dam (and then reared by a WT dam) were aggressive; OXT KO males born to heterozygous dams were not aggressive. This result raises the possibility that prenatal maternal OXT binding to fetal peripheral OXTR is an important developmental regulator of male aggression. OXT in biofluids such as urine, saliva, amniotic fluid, and blood, have the potential to act on peripheral OXTR in developing mice [11–13, 29, 30]. Further investigation into the developmental role of peripheral sensory OXTR in aggression and developmental contributions of maternal OXT is warranted.

Altogether, these data implicate OXTR in *Avil* expressing cells in adult social behaviors. The role of OXTR in regulating specific social behaviors by acting in various Avil cell populations remains to be disentangled considering that *Avil* expression has been reported in both the somatic and autonomic PNS and tissue targets, as well as the dorsal habenula of the epithalamus and trigeminal nuclei of the brainstem [19, 31–34]. While no OXTR expression has been reported in the habenula, OXTRs are in the trigeminal nuclei of the brainstem in mice and other species, but a role in social behavior has not been tested yet. Knocking out OXTR from *Avil* positive cells may alter brain region-specific developmental trajectories to alter social behavior, through developmental diaschisis. We recently reported that full body congenital OXTR KO mice have impaired *Oxt* gene expression in the paraventricular nucleus of the hypothalamus beginning in the second postnatal week which persists in males into adulthood [35]. Experience-dependent peripheral OXTR activity during development may entrain the hypothalamus to produce and release OXT in social contexts and set up the brain potential for adult social salience detection [36]. Investigating these mechanisms in humans including the conservation or diversity of OXTRs in cells of neural crest origin has translational significance.

## Materials and methods

### Experimental model and subject details

All procedures were conducted after approval by the Institutional Animal Care and Use Committee of Florida State University. All mice had *ad libitum* access to standard rodent chow (LabDiet, PMI Nutrition International, LLC, Brentwood, MO) and water. For all subjects, the day of birth was considered postnatal day 0 (P0). Experimental mice were bred in house and weaned and ear tagged at P21. Upon weaning, a small tail snip (<1mm) was collected for genotyping and males and females were housed with same sex siblings until testing at 8–10 weeks old.

Experimental subjects were adult male and female offspring from crosses of dams with the *Oxtr* gene flanked by *LoxP* recombinase binding sequences (*fxOXTR*) [8] at both alleles (*fxOXTR*^*fx/fx*^) and sires that were hemizygous for *fxOXTR* (*fxOXTR* ^*fx/+*^) and *Avil*-Cre recombinase [31] transgene positive (heterozygous for the conditional deletion of *Oxtr* from *Avil* expressing cells; OXTR^*Avil*^ HET). Male and female experimental subjects were selected from *fxOXTR*^*fx/fx*^ offspring that were either *Avil*-Cre- (*fxOXTR*^*fx/fx*^;*Avil*-Cre-; referred to as OXTR^*Avil*^ WT) or *Avil*-Cre+ (*fxOXTR*^*fx/fx*^; *Avil-Cre+*; referred to as OXTR^*Avil*^ KO).

There were 20 OXTR^*Avil*^ WT males, 15 OXTR^*Avil*^ KO males, 19 OXTR^*Avil*^ WT females, and 15 OXTR^*Avil*^ KO females tested for sociability and preference for social novelty. Only males were tested for aggression in the RIT. One OXTR^*Avil*^ WT male that went through sociability and preference for social novelty testing died from malocclusion before the RIT. To rule out a deleterious effect of the Cre-recombinase transgene on social behaviors, male and female *Avil*-Cre- and *Avil*-Cre+ littermates were also tested. Mice for these control experiments were generated by breeding wild-type (WT) dams with *Avil-*Cre+ sires. Twelve *Avil*-Cre- and 17 *Avil*-Cre+ control males as well as 20 *Avil*-Cre- and 16 *Avil*-Cre+ control females were tested.

Stimulus mice were C57BL/6J male and female mice received from Jackson Labs at approximately 5–7 weeks old and allowed to habituate to our mouse colony for at least 2 weeks prior to testing. Stimulus animals were ear tagged for identification purposes. After testing, stimulus mice were placed into a temporary holding cage to avoid social transmission of the test to cage mates that had not gone through testing yet. Stimulus animals were placed back together into their home cages after testing was completed for the day.

#### Genotyping

Tail samples were genotyped via polymerase chain reaction (PCR) to determine *fxOXTR* and *Avil-cre* genotypes. The *fxOXTR* forward primer 5’-GCT GAG TCT TGG AAG CAG GA and reverse primer 5’-GGT ACC TCC TTT GAG CTT CTG generated a 375 bp product for the *fxOXTR*^*fx/fx*^ genotype and a 305 bp product for the *fxOXTR*^*+/+*^ genotype. Heterozygous mice (*fxOXTR*^*fx/+*^) produced both PCR products. PCR thermal cycling conditions for *fxOXTR* were as follows: 94°C for 2 minutes followed by 10 cycles of 94°C for 20 seconds, 65°C for 15 seconds for the first cycle with a 0.5°C decrease per cycle, and 68°C for 10 seconds and finally 28 cycles of 94°C for 15 seconds, 60°C for 15 seconds, and 72°C for 10 seconds, followed by 1 cycle of 72°C for 2 minutes. The *Avil-cre* forward primer (5’‐GCA CTG ATT TCG ACC AGG TT) and reverse primer (5’‐GAG TCA TCC TTA GCG CCG TA) generated a product approximately 420 base pairs for transgene positive and no band for transgene negative. PCR thermal cycling conditions for *Avil-cre* were: 95°C for 2 minutes followed by 10 cycles of 95°C for 30 seconds, 65°C for 30 seconds for the first cycle with a 1°C decrease per cycle, and 72°C for 30 seconds and finally 35 cycles of 95°C for 30 seconds, 55°C for 30 seconds, and 72°C for 30 seconds, followed by 1 cycle of 72°C for 10 minutes. Post-PCR reactions were visualized by gel electrophoresis using a 2% agarose gel at 140 volts for 20 minutes for *fxOXTR* genotype determination and a 1% agarose gel at 140 volts for 20 minutes for *Avil-cre* identification. PCR products were visualized by ethidium bromide assisted imaging. All PCRs and gels were run with appropriate positive, negative, and no template controls.

#### Conditional knock-out validation

To validate the selective deletion of *Oxtr* in our Cre-LoxP strategy, we compared the expression levels of *Oxtr* mRNA in brain and peripheral tissues of adult OXTR^*Avil*^ WT (n = 2) and OXTR^*Avil*^ KO mice (n = 2). Specifically, we dissected olfactory bulbs and piriform cortex as representative neural tube derived central nervous system tissue, and trigeminal ganglia as a representative neural crest derived peripheral nervous system tissue. Because *Oxtr* is expressed at low levels, to calibrate the expected difference between WT and KO, we also compared the expression level of *Oxtr* mRNA in olfactory bulbs and piriform cortex from WT mice (n = 2) to conventional OXTR KO [4, 11, 35] mice (n = 2) from archived tissue. Animals were euthanized by CO_2_, tissues were extracted and immediately fresh frozen. Samples were stored at -80°C until RNA extraction by the Trizol method. RNA quality was confirmed by gel electrophoresis and quantity was measured by Nanodrop. Total RNA was DNAse digested and reverse transcribed. RT-qPCR was performed with primers for *Gapdh* (reference gene; *Gapdh* forward 5’-AAT GGT GAA GGT CGG TGT G and *Gapdh* reverse 5’-GTG GAG TCA TAC TGG AAC ATG TAG; 150bp product covering nucleotides 92–241 of NM_001289726.1), *Oxtr* (*Oxtr* forward 5’-CGC ACA GTG AAG ATG ACC TT and *Oxtr* reverse 5’-ATG GCA ATG ATG AAG GCA GA; 131bp product covering nucleotides 839–969 of NM_001081147.2) and *Avil* (conditional reference gene; *Avil* forward 5’- CTA AAG AAC GAG CTG GGA GAT and *Avil* reverse 5’- GGA TTC AGG TAA AGT GTT GCG; 80bp product covering nucleotides 2315 to 2374 of NM_009635.3). SYBR dye was used in a cycling program: 94°C 5 minutes, 40 cycles of 94°C for 30 seconds, 60°C for 30 seconds, 55°C for 5 seconds. Melt curve analysis was used to confirm the appearance of a single amplicon. Non DNAse-treated RNA templates for each sample were used to confirm the absence of genomic DNA contamination and water was used as a no template control. Both controls performed as expected (zero to negligible amplification). These data are available in the S1 Data File.

#### Behavioral testing

In all procedures, the experimenter wore a disposable lab coat, gloves, and a facemask. Tests were conducted between 9am and 5pm with mice housed on a standard 12:12 light cycle with lights on at 8am. Mice were housed in a single room in cages with wire tops and wood chip bedding. Cages were cleaned once per week. Both male and female adults were tested in the sociability and preference for social novelty test. Males only were tested in the RIT after 2 weeks of social isolation. Fig 1 illustrates the experimental timeline.

#### Sociability and preference for social novelty tests

The sociability and social preference tests are conducted in a three-chambered testing apparatus described by Moy et al., (2004), wherein a rectangular testing apparatus is equally divided into two outer chambers and a center chamber [37]. During the sociability test, one side chamber contains a conspecific contained in a wire cup, or holding tower, and the opposite side chamber contains an empty wire cup. The time a subject chooses to spend in each chamber is measured after being placed into the center chamber. The proportion of time spent in the chambers is used as an index of sociability of the mouse because a highly social mouse will spend more time in the chamber with a conspecific over the empty chamber compared to a non-social mouse. The purpose of containing the stimulus mouse in a cup or holding tower is to allow the subject to approach the conspecific and minimize the conspecifics behavior potentially confounding the subject’s behavior, which is the primary variable of interest. The preference for social novelty test is conducted directly after the sociability test, wherein a new conspecific is placed in the empty chamber opposite the chamber containing the now familiar mouse used in the sociability test. If the time spent in the chamber with the new stranger conspecific is higher than the time spent in the chamber with the familiar conspecific, then it is inferred that the subject prefers social novelty. C57BL/6J adult mice have been shown to spend more time in the chamber with a same sex conspecific compared to the empty chamber in the sociability test and more time in the chamber with a stranger versus familiar conspecific in the preference for social novelty test in several studies [4, 6, 7], with some exception that may reflect a conflation of social familiarity and contextual cues [38].

Behavioral testing was conducted in the three-chambered social apparatus (catalog # 46503, Ugo Basile). The dimensions of each chamber were 20 × 40 × 22 cm(h). The walls of the chamber were made of clear Perspex. The bottom of the apparatus consisted of a non-reflective gray colored floor. Two sliding doors, also made of clear Perspex, were 5 × 8 cm(h) and when in place confined the subject to the center chamber. The top and bottom of the two-cylinder shaped stimulus holding towers were made of gray PVC and the grid bars were metal, 25 cm tall, 3 mm in diameter, and spaced 7 mm apart. Two lids made of clear Perspex with holes were placed on the outermost chambers to prevent the subject from climbing on top of the stimulus towers and escaping the apparatus during the test. Subjects were isolated for at least 1 hour in the testing room prior to testing in standard cages identical to those used in housing, with access to food and water. Prior to testing, the entire three chambered apparatus was wiped down with soft absorbent paper towels and a 10% bleach solution in filtered, deionized water and then a final wipe with sufficient water to remove residual odor before finally being dried with paper towels. Prior to testing, holding towers were also sprayed and wiped down with 10% bleach, then water, and then dried using soft absorbent paper towels. The subject habituated to the testing apparatus with the doors to the outermost chambers opened for 10 minutes. At the end of the 10-minute habituation period, an empty holding tower was placed in one outer chamber and a holding tower containing an age-, sex- and weight- matched conspecific was placed in the opposite chamber. The sociability test lasted for 10 minutes. The social novelty test then immediately commenced by placing an unfamiliar age-, sex-, and weight- matched conspecific into the previously empty tower. The social novelty test lasted for 10 minutes and included a familiar conspecific in one chamber (from the sociability test) and the unfamiliar conspecific in the opposite chamber. The familiar and unfamiliar stimulus animals were housed separately prior to arrival in our colony and had never interacted with each other or with the subjects. Stimulus animals were kept in different rooms from the subject mice on the day of testing. Stimulus mice were habituated to the holding towers and testing apparatus for at least three days prior to testing for 40 minutes per day. Stimulus mice were used once a day for a maximum of two days.

#### Resident intruder test

In the RIT, an “intruder” or male stranger is placed into the home cage of an adult “resident” male that has been socially isolated or has had mating experience. Aggressive behaviors are measured including attack duration and frequency as well as latency to first attack. Only males were tested in the RIT. After the preference for social novelty test was completed, virgin male subjects were singly housed for two weeks before the RIT. Subjects’ cages were not cleaned for at least a week prior to the RIT (ranging from 7–13 days). On the day of the RIT, subjects’ home cages were transferred to the testing room at least 1 hour prior to testing (range was 1–2 hours). The RIT commenced when a male intruder weighing slightly less than the subject was placed into the subject’s (resident) home cage. The test lasted 15 minutes and ended with the intruder being removed from the home cage of the resident. A researcher monitored the entire RIT to prevent possible serious injuries to the intruder if necessary. Because we did not pre-screen intruders for absence of aggression, to analyze the effects of resident genotype on aggression, we only analyzed data from tests where the C57BL/6J stimulus intruder did not initiate aggression in the RIT. This was determined by scoring instances of the intruder starting a clinch attack without knowledge of resident genotype. After removing subjects with intruder-initiated aggression from the dataset, we analyzed data from 14/19 OXTR^*Avil*^ WT and 13/15 OXTR^*Avil*^ KO males and 8/12 *Avil*-Cre- and 10/17 *Avil*-Cre+ males.

### Quantification and statistical analysis

#### Behavior quantification

All tests were video recorded with cameras positioned to achieve a bird’s eye view and side views of the entire testing apparatus. Experimenters blind to the genotype of the subjects later scored behaviors using JWatcher software (UCLA). For the sociability and preference for social novelty tests, the duration of time spent in each chamber, and the duration and frequency of tower sniffing were quantified. In addition, tower approach latencies were measured and defined as the time it took the subject from the start of the test to approach a tower. For the RIT, the latency, duration and frequency of clinch attacks, as well as social (including the sum of anogenital, head, and body sniffing, chasing, and licking and grooming) and non-social (the sum of self-grooming, nest building, running, and resting) behaviors were scored. These data are available in the S1 Data File.

#### Statistics

To determine if genotype influences the ability to display sociability and social novelty preferences, we analyzed tower investigations, chamber durations, and tower approach frequencies with a repeated measures two-way (sex and genotype) analysis of variance (RM ANOVA). Data for the sociability and preference for social novelty tests are presented as individual data points with averages ± standard error of the mean and as difference scores, in line with other studies [6, 20], to compare sociability degree. Difference scores for investigation (sniff) duration, chamber duration, and approach frequency were each calculated by subtracting the performance towards the empty tower/chamber from the stimulus mouse in the sociability test. A positive difference score reflects sociability. Similarly, social novelty preference difference scores were computed for investigation (sniff) duration, chamber duration, and approach frequency by subtracting the performance toward the familiar from the novel conspecific. A positive difference score reflects preference for the novel conspecific. Latencies to approach stimulus towers were analyzed with two-way ANOVAs. For the RIT, behavioral variables were compared between genotypes with linear mixed models due to non-normal data distributions. All data were checked for normality and homogeneity of variance before running analyses. If normality or homogeneity of variance were violated, data were first transformed to meet assumptions for parametric tests or analyzed with a non-parametric test where noted. The data were also checked for outliers. The total time spent sniffing the empty and conspecific containing towers were investigated for outliers based off Q-Q plots, histograms, and greater than 1.5 interquartile ranges from the grand mean. Thus, 3 OXTR^*Avil*^ WT males from the same litter (indicating a potential litter effect on atypical, low social investigation), 1 OXTR^*Avil*^ KO male and 1 OXTR^*Avil*^ KO female with low total tower investigation times ranging from 0–37 seconds over the 10-minute test were removed from all further analyses for the sociability and social novelty tests. Importantly, removing these outliers resulted in homogeneity of variance and a normal distribution and did not change the outcome of significant test results but met the required variance and distribution parameters for confident interpretation of parametric tests. In addition, the low investigation duration of the removed outliers questions the validity of their test results since they had minimal participation. Tower approach latencies were square root transformed to reach normality and 1 *Avil*-Cre+ female outlier was removed with a latency of 566 seconds to approach the empty tower and 23 seconds to approach the tower with the unfamiliar mouse. 2 *Avil*-Cre+ males, 1 *Avil*-Cre- female, and 1 *Avil*-Cre+ female were identified as outliers based on deviation from the grand mean of total tower sniffing in the sociability test and removed from further sociability and preference for social novelty analyses. These subjects spent a range of 3–17 seconds sniffing both towers combined during the entire 10-minute test. Statistical analyses were conducted in SPSS (Version 25.0) and details can be found in the results section text and S1 Table in S1 File.

To compare our results with published studies, we estimated effect sizes (Cohen’s *d*) from prior work by extracting means and SEMs (converted to standard deviations) from plots and entering this information in the effect size calculator at https://lbecker.uccs.edu/.

## Supporting information

S1 File This file contains additional supporting figures and a statistical results table for all analyses performed. (DOCX)

S1 Data This file contains the original data reported in this study. (XLSX)
