# Supplementary material for: Oxytocin receptor disruption in Avil-expressing cells results in blunted sociability and increased inter-male aggression
Source: PLoS One. 2021 Nov 30;16(11):e0260199. doi: 10.1371/journal.pone.0260199 (PMC8631681; doi:10.1371/journal.pone.0260199)
Supplement: S1 File — (DOCX) [file pone.0260199.s001.docx]

**Supporting information**

Supplemental Material for:

Oxytocin receptor disruption in *Avil-*expressing cells results in blunted sociability and increased inter-male aggression.

Manal Tabbaa^1^, Ashley Moses^1^, and Elizabeth A. D. Hammock^1^*

^1^Department of Psychology and Program in Neuroscience, The Florida State University, Tallahassee, FL, United States of America

*corresponding author

E-mail: [ehammock@fsu.edu](mailto:ehammock@fsu.edu) (EADH)


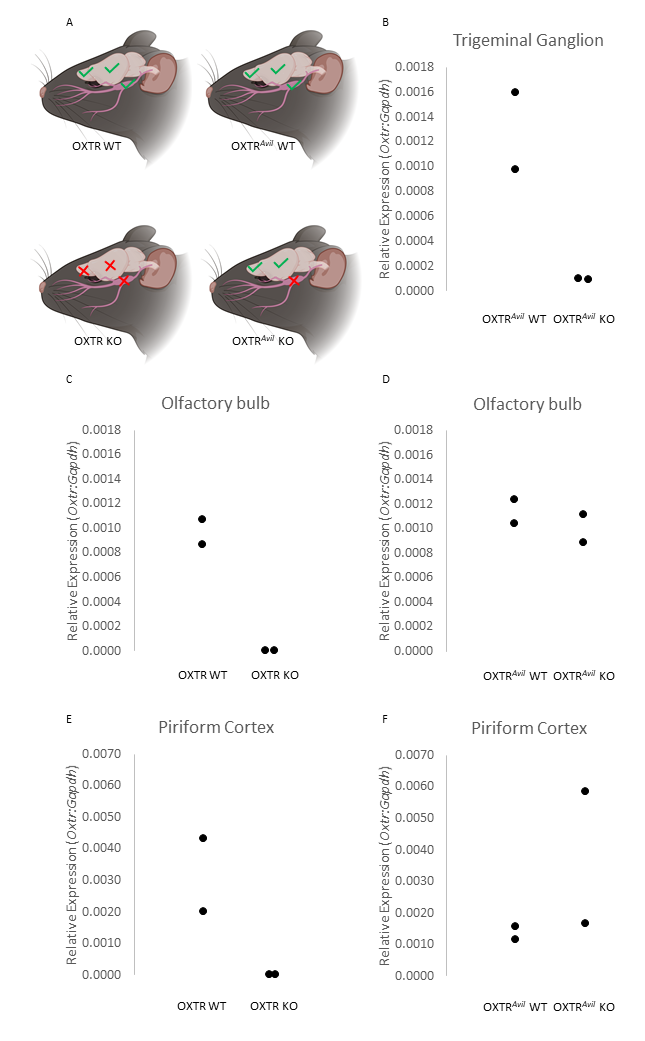


**S1 Fig.** **RT-qPCR validation of successful conditional deletion of *Oxtr* in OXTR*^Avil^* KO line.** A) Expected expression patterns (green check = expression expected; red X = expression not expected) of *Oxtr* in the neural crest derived *Avil*-expressing trigeminal ganglion and non-neural crest derived *Avil*-non-expressing olfactory bulb and piriform cortex in wild-type mice, conventional OXTR KO mice, and conditional OXTR KO mice (OXTR*^Avil^* KO). B) *Oxtr* mRNA was detectable from trigeminal ganglia dissected from adult wild-type (OXTR*^Avil^* WT, n=2), but not conditional knock-outs (OXTR*^Avil^* KO, n=2). C) Loss of *Oxtr* mRNA expression was evident when comparing the olfactory bulbs of conventional global OXTR KO (n=2) to OXTR WT mice (n=2). D) In contrast, *Oxtr* mRNA was still present in the olfactory bulb (a tissue that does not express *Avil* and is not neural crest-derived) of OXTR*^Avil^* KO (n=2), just as it was expressed in OXTR*^Avil^* WT mice (n=2). E) Within the brain, *Oxtr* mRNA expression was lost in conventional OXTR KO (n=2) compared to OXTR WT (n=2) as measured in the piriform cortex. F) *Oxtr* mRNA expression in the piriform cortex was not reduced in OXTR*^Avil^* KO (n=2) compared to WT (n=2). Cartoon of mouse head created with Biorender.com.

**S2** **Fig.** **The presence of the *Avil*-Cre transgene does not impair sociability.** There were no differences between *Avil*-Cre- and *Avil*-Cre+ males and females in latencies to approach the empty tower (white markers) compared to the tower holding the unfamiliar stimulus mouse (stimulus tower; gray markers; A). There were no differences in the preference of *Avil*-Cre+ versus *Avil*-Cre- males and females for the stimulus tower over the empty tower in approach frequencies (B), tower sniffing (C), and chamber durations (D). However, a main between subject effect of genotype revealed *Avil*-Cre+ subjects to have lower overall chamber durations compared to *Avil*-Cre- subjects (D). Individual data points are graphed as well as means ± standard error of the mean. *= main effect of genotype, p < 0.05, see S1 Table.
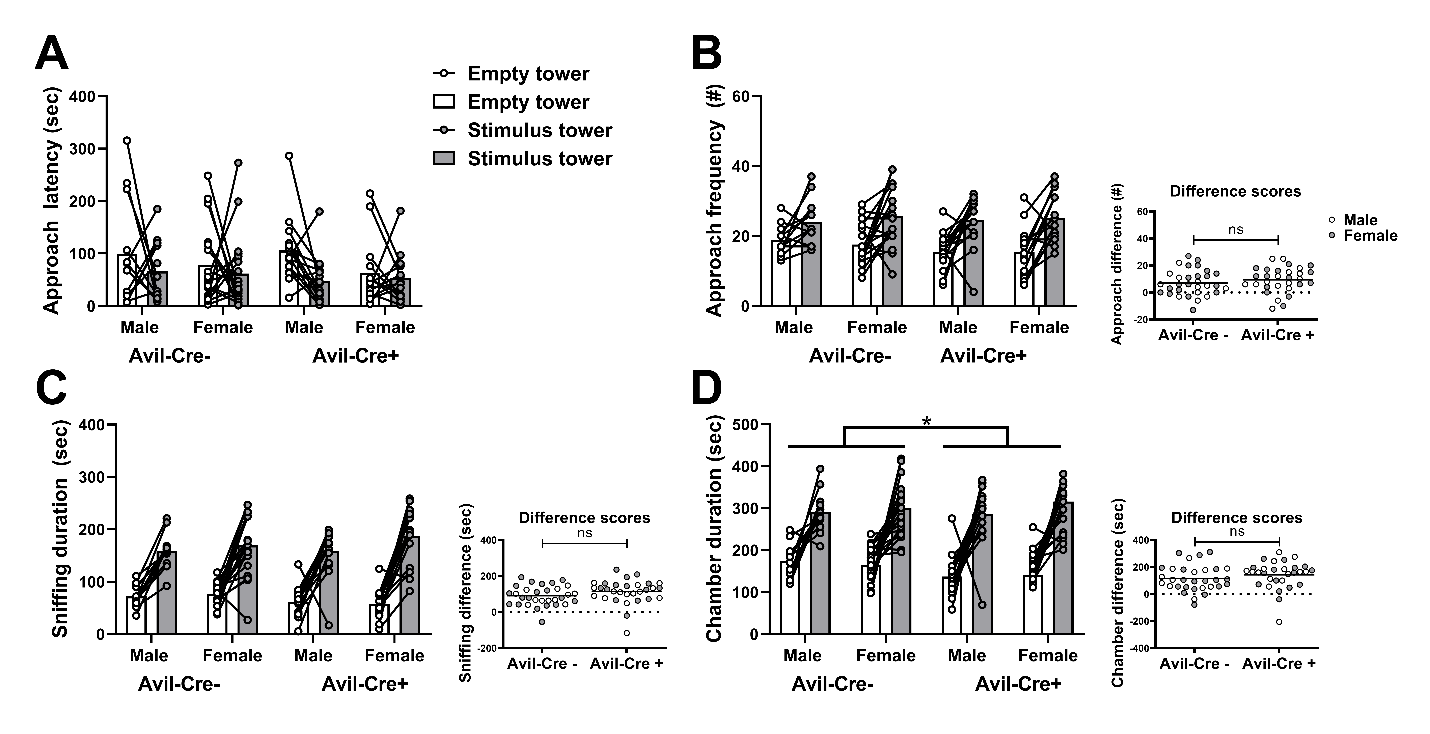


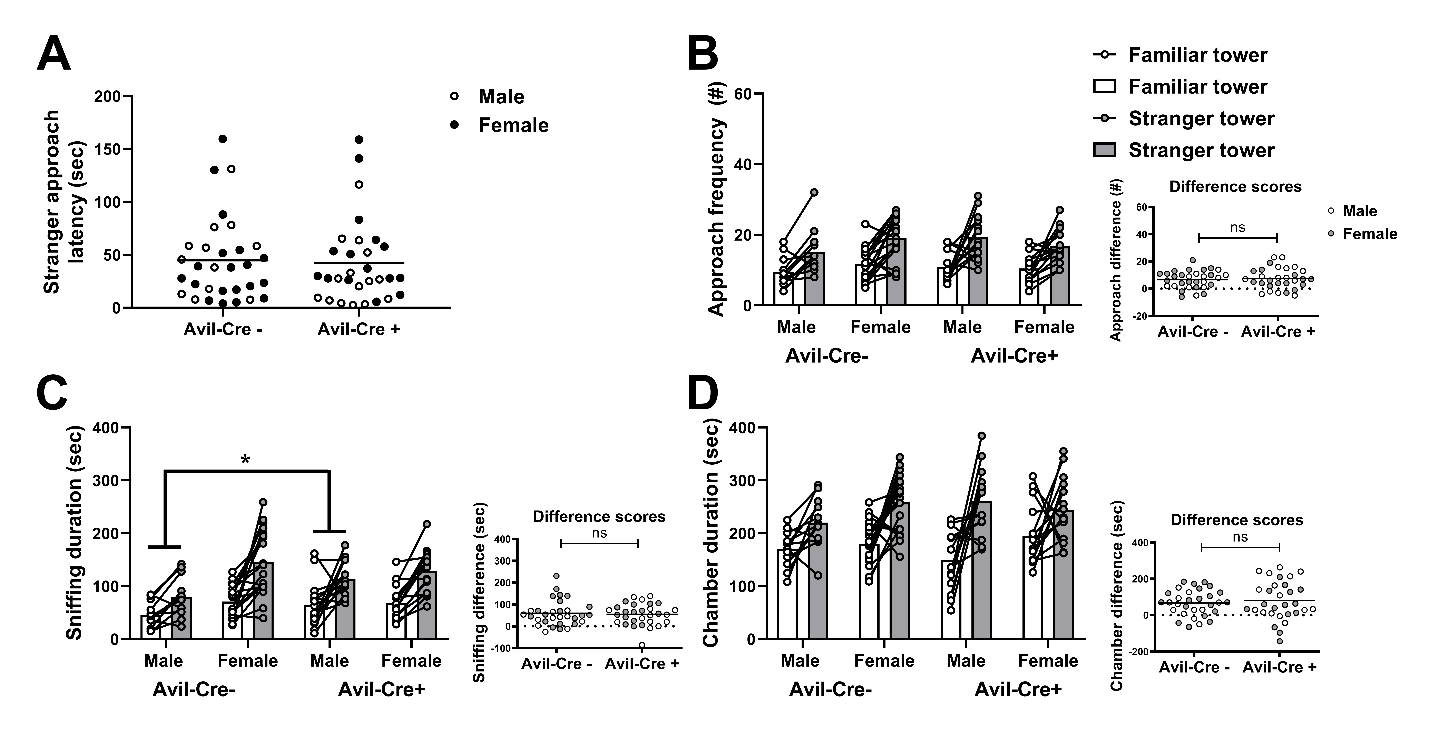


**S3 Fig. The presence of the *Avil*-Cre transgene does not impair social novelty preference.** There were no differences between *Avil*-Cre- and *Avil*-Cre+ males and females in latencies to approach the tower containing the stranger mouse (A). There were no differences in the ability of *Avil*-Cre+ versus *Avil*-Cre- males and females to differentiate a stranger mouse (stranger tower; gray markers) from the familiar mouse (familiar tower; white markers) in tower approach frequencies (B), time spent sniffing the towers (C), and chamber durations (D). However, *Avil*-Cre+ males had increased tower sniffing durations compared to *Avil*-Cre- males (C). Individual data points are graphed as well as group averages. *= main effect of genotype in males, adjusted p < 0.05, see S1 Table.


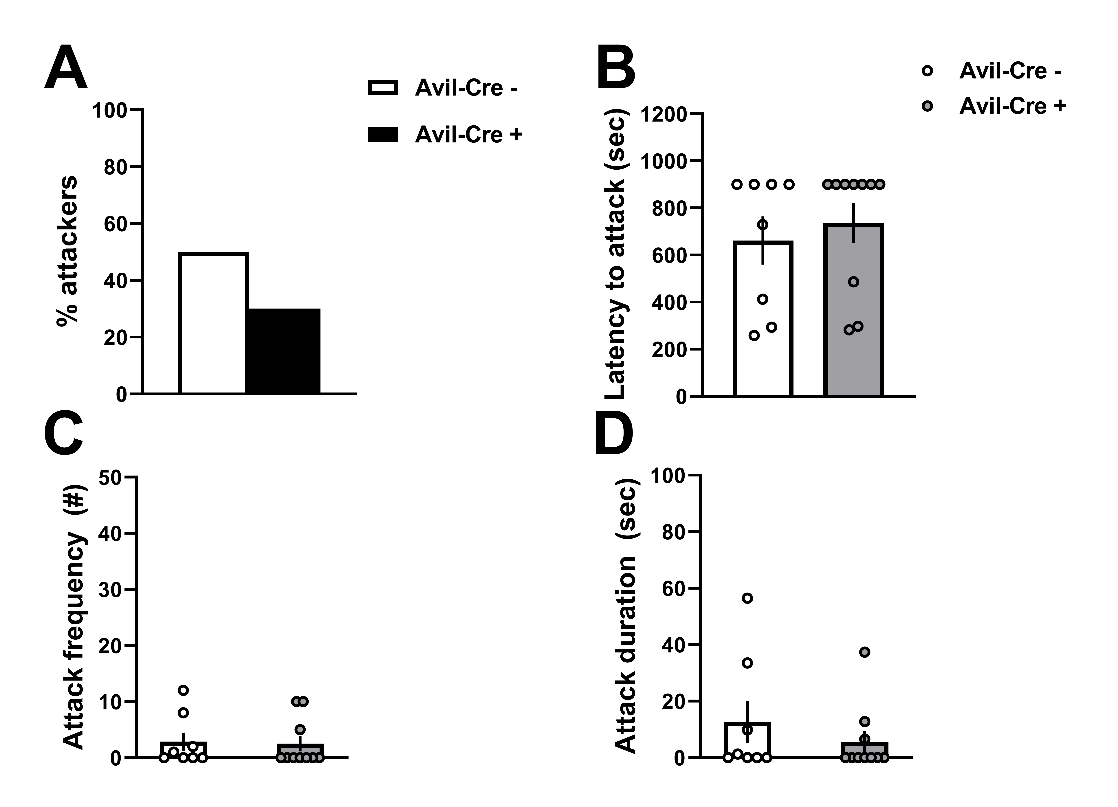


**S4 Fig. The presence of the *Avil*-Cre transgene alone does not increase aggression.** There were no differences between *Avil*-Cre- and *Avil*-Cre+ males in the percentage of attackers (A), latencies to attack (B), attack frequencies (C), and attack durations (D) in the RIT. Individual data points are graphed as well as group means ± standard error of the mean, see S1 Table.


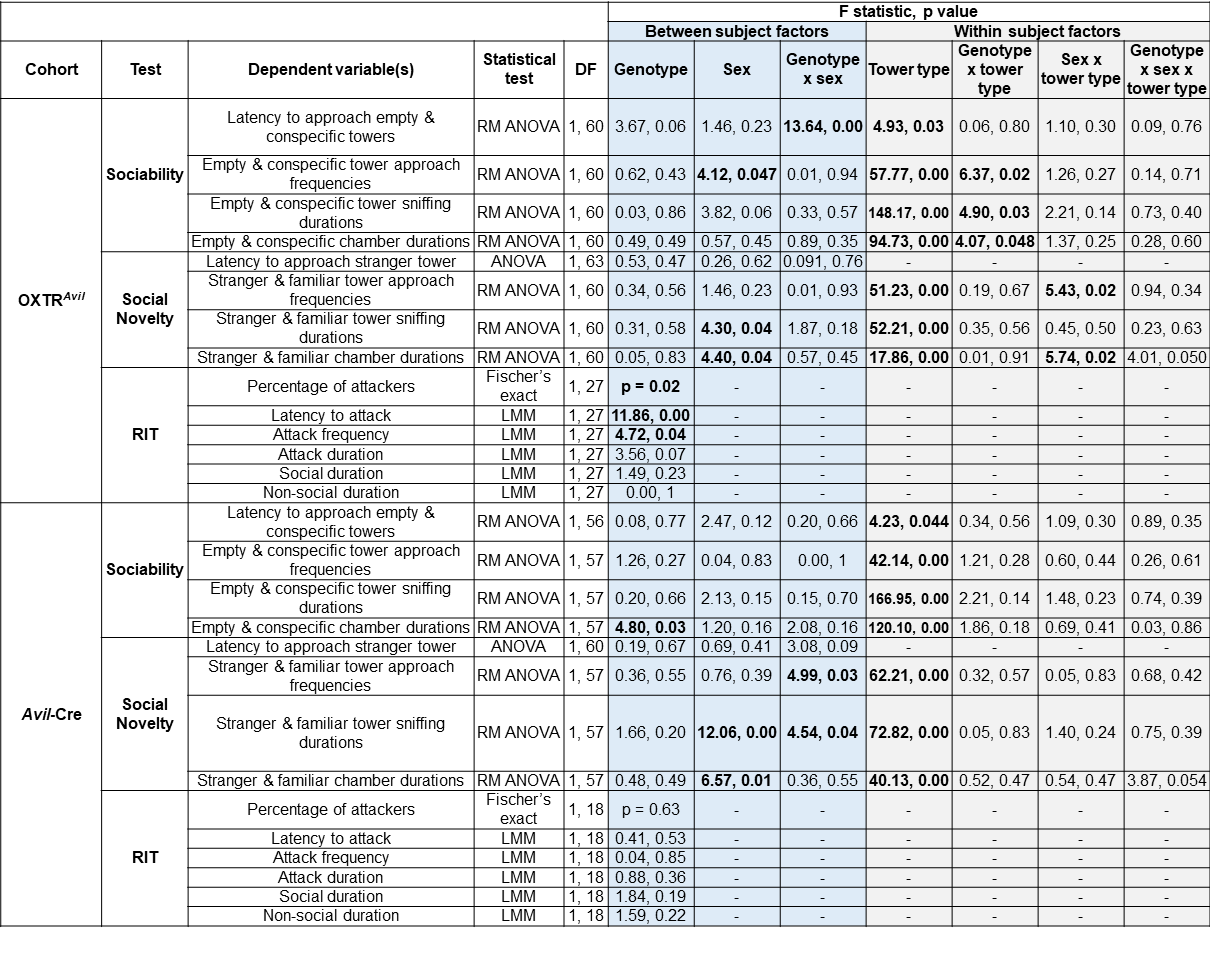


**S1 Table. Statistical details for all analyses in this study.** Significant results are bolded. DF = Degrees of Freedom; RIT = Resident Intruder Test; RM ANOVA = Repeated Measures Analysis of Variance; LMM = Linear Mixed Model
